# Multidimensional oral health and frailty in older adults: A sex-stratified cross-sectional study

**DOI:** 10.1371/journal.pone.0360682

**Published:** 2026-10-16

**Authors:** Jeong-Eun Yang, SeolHwa Moon

**Affiliations:** 1 Department of Nursing, Jesus University, Jeonju, Jeonbuk State, Republic of Korea; 2 Department of Nursing, Hoseo University, Asan, Chungcheongnam-do, Republic of Korea; Universidade Federal Fluminense, BRAZIL

## Abstract

**Background:**

Frailty in older adults is a critical public health concern associated with increased morbidity, disability, and healthcare utilization. Although systemic health and socioeconomic factors are well-established determinants of frailty, the multidimensional nature of oral health has received relatively limited attention in previous research. The aim of this study was to examine the associations between general characteristics, health-related factors, oral health factors, and frailty among community-dwelling older adults, and to identify sex differences in frailty.

**Methods:**

This cross-sectional study used data from the 2022 Korean Longitudinal Study of Aging (KLoSA) and included 3,729 individuals aged 65 years and older. Frailty was categorized as robust, pre-frail, or frail using the Frailty Instrument developed for older Korean adults. General characteristics, health-related factors, and oral health-related factors (structural, behavioral, and subjective) were assessed through structured questionnaires. Hierarchical logistic regression analysis was performed in three steps to identify factors associated with frailty.

**Results:**

Of the participants, 16.0% were frail and 84.0% were non-frail (31.5% pre-frail and 52.5% robust). Sex-stratified analyses also showed that structural oral health factors were most strongly associated with frailty in both sexes. Edentulous status without denture use was associated with the highest odds of frailty in both women (OR = 3.76, 95% CI = 3.72–3.80) and men (OR = 2.95, 95% CI = 2.91–2.98). In contrast, sex differences were observed for some subjective oral health factors: tooth sensitivity was associated with higher odds of frailty in women (OR = 1.82, 95% CI = 1.80–1.84) but lower odds in men (OR = 0.85, 95% CI = 0.84–0.86).

**Conclusions:**

Oral health conditions, including untreated tooth loss, difficulty in chewing or swallowing, and oral pain, were significantly associated with frailty. These findings suggest that oral health may be an important factor to consider in relation to frailty among community-dwelling older adults.

## Introduction

As population aging accelerates globally, maintaining functional ability and improving the quality of life for older adults has become a major public health challenge. The World Health Organization (WHO) defines healthy aging as the process of maintaining and enhancing functional abilities [1]. However, many older adults are at risk of losing their independence due to chronic diseases, physical decline, and cognitive changes [2]. In this context, identifying factors that influence functional health in later life is essential.

Oral health has increasingly been recognized as an important determinant of overall health in older adults. Rather than simply reflecting the absence of disease, oral health is a multidimensional concept that includes structural conditions, functional abilities, and psychosocial aspects, including speaking, chewing, swallowing, and social interaction [3]. It can be categorized into structural factors (e.g., number of natural teeth and use of dentures), oral health-related behaviors (e.g., dental care), and subjective oral health factors (e.g., chewing and swallowing function, oral pain, tooth sensitivity). Several of these factors have been shown to be associated with frailty older adults [4,5].

Poor oral health may affect overall well-being through several pathways. For example, reduced chewing ability can lead to decreased food intake and nutritional imbalance, while oral discomfort may limit social participation and daily activities [6,7]. Previous studies have reported that various oral health-related factors, such as tooth loss, chewing difficulties, and swallowing difficulties, are significantly associated with frailty [4,7,8]. In addition, tooth loss is also associated with chronic diseases, demonstrating a complex interaction between oral health and systemic health [9]. Frailty is a common geriatric syndrome characterized by reduced physiological reserve and increased vulnerability to external stressors [3,10]. It is associated with adverse outcomes, such as falls, disability, hospitalization, and death [3,11]. Because frailty is influenced by various factors, it is important to identify modifiable relevant to its prevention and management [2,12]. Although several studies have investigated the relationship between oral health and frailty, most have focused on specific indicators such as tooth loss or chewing function. Thus, limited evidence is available on the relationship between frailty and multiple dimensions of oral health considered comprehensively, including structural, behavioral, and subjective factors. As a result, there is a lack of understanding regarding how various aspects of oral health are comprehensively related to frailty.

In addition, the association between oral health and frailty may vary by sex. Men and women differ in biological characteristics, health behaviors, and patterns of healthcare utilization, which may influence how oral health is related to health outcomes [13,14]. For instance, women are generally more likely to engage in preventive health behaviors, whereas men tend to seek care less frequently at a later age [14]. However, studies that consider these differences in the context of oral health and frailty remain limited.

Therefore, this study aimed to investigate the association between multidimensional oral health factors and frailty among community-dwelling older adults. Specifically, structural, behavioral, and subjective aspects of oral health were considered together, and potential sex differences were explored. By providing an integrated understanding of the relationship between oral health and frailty, this study may help inform approaches to addressing oral health and frailty in older adults.

## Materials and methods

### Study design

This descriptive survey study used secondary data analysis to identify the level of frailty among community-dwelling older adults and to determine the factors associated with frailty. Data from the 9th wave (2022) of the Korean Longitudinal Study of Aging (KLoSA) were used [15]. The data were accessed for research purposes on September 6, 2024. This study was conducted in accordance with the Strengthening the Reporting of Observational Studies in Epidemiology (STROBE) guidelines.

### Participants

A secondary analysis was conducted using raw data from the 9th wave of the KLoSA. KLoSA is a nationally representative longitudinal survey conducted biennially by the Korea Employment Information Service since 2006 and provides foundational data for policy development by assessing the social, economic, psychological, and health status of older adults. Of the 6,057 participants in the 9th wave, those younger than 65 years (n = 1,464) and those with missing grip strength data (n = 864) were excluded. Consequently, 3,729 participants were included in the final analysis.

### Variables

The study variables were derived from the KLoSA survey data and were primarily obtained through self-report. Accordingly, they reflect perceived health status rather than objective clinical measures. The variables were classified into general characteristics, health-related factors, and oral health-related factors.

1) General Characteristics

General characteristics included age, sex, educational level, living arrangement, and economic satisfaction. Age was treated as a continuous variable, and the analysis included only participants aged 65 years or older. Sex was categorized as male or female. Educational level was classified as elementary school or lower (≤ 6 years of education) and middle school or higher (≥ 7 years of education). Living arrangement was categorized as living alone for single-person households and not living alone for households with two or more members. Economic satisfaction was measured using a quality-of-life item assessing satisfaction with one’s economic status relative to that of peers. Responses ranged from 0 to 100, with higher scores indicating greater economic satisfaction, and the variable was analyzed as a continuous measure.

2) Health-related factors

Health-related factors included perceived health status, functional dependence, number of chronic diseases, and body mass index (BMI). Perceived health status was measured using a single item asking participants to rate their current health status. Responses were recorded on a 5-point scale and categorized into good (“very good” or “good”) and poor (“average,” “poor,” or “very poor”). Functional dependence was assessed using the Korean Instrumental Activities of Daily Living (K-IADL) scale [16]. The K-IADL includes items on telephone use, financial management, medication use, transportation use, and household chores, with higher scores indicating greater dependence on instrumental activities of daily living. The K-IADL score was analyzed as a continuous variable. The number of chronic diseases was calculated by summing physician-diagnosed conditions among 10 diseases, including cerebrovascular disease, heart disease, liver disease, chronic lung disease, cancer, diabetes, hypertension, mental illness, osteoarthritis or rheumatism, and dementia. BMI was calculated from self-reported height and weight as weight (kg) divided by height squared (m²).

3) Oral health-related factors

Oral health-related factors were classified into structural factors, oral health behavior, and subjective oral health factors based on previous studies [7,9,17].

- Structural oral health factors were derived from the number of natural teeth and denture use. The number of natural teeth was assessed by asking participants how many teeth they currently retained, including wisdom teeth. Denture use was determined based on whether participants regularly wore dentures. Based on previous studies on masticatory function, the number of natural teeth was categorized using a cutoff of 20 teeth [18,19] into three groups: 21 or more teeth, 1–20 teeth, and edentulous. These categories were further combined with denture use to form five structural oral health groups: 21 or more teeth, 1–20 teeth with dentures, 1–20 teeth without dentures, edentulous with dentures, and edentulous without dentures.
- Oral health behavior was measured based on dental care utilization within the previous 2 years. Participants were classified as having utilized dental care if they had visited a dental clinic at least once during that period and as not having utilized dental care if they had not. This classification reflects the biennial survey cycle of the KLoSA data.
- Subjective oral health factors were measured using four selected items from the Korean version of the Geriatric Oral Health Assessment Index (K-GOHAI) reflecting oral function and symptoms. Masticatory function was assessed using an item asking whether participants had difficulty chewing hard foods, such as apples or meat. Additional items assessed dysphagia, oral pain, and tooth sensitivity. All items were rated on a 6-point scale ranging from never to always. For the analysis, responses of “always,” “very often,” and “often” were categorized as indicating the presence of the relevant symptom or functional limitation, whereas responses of “sometimes,” “rarely,” and “never” were categorized as indicating its absence. Accordingly, all variables were treated as dichotomous variables. Although dichotomization may result in loss of information and reduced statistical power, it remains widely used in health research because it facilitates interpretation and helps distinguish clinically meaningful groups [20,21].

4) Frailty

Frailty, the dependent variable of the present study, was assessed using the Frailty Instrument (FI). The FI reflects both physical and psychosocial dimensions and comprises three components: exhaustion, social isolation, and weakness [22]. Exhaustion was assessed using self-reported items on fatigue or difficulty in performing daily activities during the previous week, whereas social isolation was measured based on participation in social activities. Weakness was assessed using grip strength according to sex-specific criteria. Each item was assigned 1 point if the criterion was met and 0 points otherwise, and the total score ranged from 0 to 3. Based on the total score, frailty was classified as robust for 0 points, pre-frail for 1 point, and frail for 2 points or higher [23].

### Statistical analysis

Data were analyzed using IBM SPSS Statistics version 25.0 with KLoSA sample weights. Participants’ characteristics and frailty status were summarized using weighted descriptive statistics. Differences in frailty according to general, health-related, and oral health-related factors were examined using weighted univariate logistic regression. Factors associated with frailty were identified using hierarchical multivariate logistic regression in three steps: general characteristics, health-related factors, and oral health-related factors. Variables significant in the univariate analysis and those considered theoretically relevant based on prior research were included. Following the hierarchical conceptual framework proposed by Victora et al. [24], variables were entered sequentially based on their conceptual relevance, beginning with background characteristics, followed by health-related factors. Oral health-related factors were entered into the final step to assess their independent association with frailty. The results of the multivariate analysis are presented as odds ratios (ORs) and 95% confidence intervals (CIs), and model fit was assessed using a goodness-of-fit test. Finally, to identify sex differences in associated factors, multivariate logistic regression analyses were conducted separately by sex. To assess the robustness of the findings, a sensitivity analysis was conducted by comparing the results of the primary weighted models with those of unweighted models using the same statistical procedures. Statistical significance was set at *p* < 0.05.

### Ethical approval

The KLoSA data were collected with approval from Statistics Korea and informed consent from all participants. The KLoSA data are publicly accessible on the Employment Survey website in a de-identified format, with all personal identifiers removed, in compliance with Articles 33 (Protection of Confidentiality) and 34 (Obligations of Persons Engaged in Statistical Work) of the Statistics Act and the Personal Information Protection Act. These data are available for download to any individual who registers on the website. Because the variables analyzed in this study were obtained from pre-existing raw data within the scope of previously secured consent, no additional procedures for data collection or informed consent were necessary. This secondary analysis was determined to be exempt from review by the Public Institutional Review Board in the Republic of Korea (IRB No. P01-202410-01-016).

## Results

The general characteristics of the participants are presented in Table 1. The mean age of the participants was 73.38 years (SE = 0.01), and 55.0% were women. Among the participants, 21.3% were living alone, and the mean score for economic satisfaction was 57.89 (SE = 17.97). Regarding health-related factors, 76.0% of the participants reported poor perceived health status, and the mean number of diagnosed chronic diseases was 1.66 (SE = 0.01). In terms of oral health-related factors, 6.6% were edentulous and did not wear dentures, 29.9% had visited a dental clinic within the past 2 years, 36.3% reported difficulty chewing, 30.3% reported swallowing difficulty, 8.6% reported oral pain, and 13.3% reported tooth sensitivity. In addition, 16.0% of the total participants were classified as frail.

**Table 1:** General characteristics of the study participants (N = 3,729).

Variables	Weighted % or M ± SE	Actual range
General characteristics		
Age (years)	73.38 ± 0.01	65–100
Sex		
Female	2,115 (55.0)	
Male	1,614 (45.0)	
Education		
0–6	1,590 (35.7)	
> 6 years	2,139 (64.3)	
Living alone		
Yes	801 (21.3)	
No	2,928 (78.7)	
Economic satisfaction	57.89 ± 17.97	
Health-related factors		
Perceived health		
Good	831 (24.0)	
Poor	2,898 (76.0)	
IADL (score)	0.37 ± 0.01	0–10
Number of chronic diseases	1.66 ± 0.01	0–7
BMI (kg/m^2^),	23.74 ± 0.01	13.69–37.69
Oral health-related factors		
Structural factors		
Number of natural teeth and denture use		
> 20 teeth	2,329 (65.5)	
1–20 teeth, with denture	400 (9.2)	
1–20 teeth, without denture	430 (11.4)	
Edentulous, with denture	316 (6.7)	
Edentulous, without denture	228 (6.6)	
Oral health behavior		
Dental clinic visit (within 2years)		
Yes	1,081 (29.9)	
No	2,246 (70.1)	
Subjective oral health factors		
Chewing difficulty		
Yes	1,211 (36.3)	
No	2,518 (63.7)	
Swallowing difficulty		
Yes	1,162(30.3)	
No	2,566(69.7)	
Oral pain		
Yes	333 (8.6)	
No	3,396 (91.4)	
Tooth sensitivity		
Yes	529 (13.3)	
No	3,200 (86.7)	
Frailty status		
Robust	1,960 (52.5)	
Pre-frail	1,174 (31.5)	
Frail	595 (16.0)	

*Note*. Continuous variables are presented as weighted means ± standard errors (SE), and categorical variables as weighted percentages. Frequencies are unweighted. SE: standard error; IADL: instrumental activities of daily living; BMI: body mass index. Missing values: Dental clinic visits within 2 years (n = 2); BMI (n = 24); number of natural teeth and denture use (n = 26).

In the univariate analysis, all general and health-related factors were significantly associated with frailty. All oral health-related factors were also significantly associated with frailty, and consistent associations observed across factors of structural, behavioral, and subjective oral health. Among these, being edentulous without wearing dentures (OR = 3.81, 95% CI = 3.79–3.84) and having difficulty chewing (OR = 3.22, 95% CI = 3.21–3.24) showed the strongest associations (Table 2).

**Table 2:** Univariate logistic regression analysis for factors associated with frailty (N = 3,729).

Variables	OR (95% CI)	*p*-value
General characteristics		
Age (years)	1.12 (1.12–1.12)	< .001
Sex		
Female (ref: Male)	1.05 (1.05–1.05)	< .001
Education		
≤ 6 years (elementary school or less) (ref: > 6 years)	2.92 (2.90–2.93)	< .001
Economic satisfaction	0.96 (0.96–0.96)	< .001
Living alone (ref: No)	1.92 (1.91–1.93)	< .001
Health-related factors		
Perceived health (ref: Good)	3.58 (3.55–3.60)	< .001
IADL (score)	1.38 (1.38–1.38)	< .001
Number of chronic diseases	1.49 (1.49–1.49)	< .001
BMI (kg/m^2^)	0.93 (0.92–0.93)	< .001
Oral health-related factors		
Structural factors		
Number of natural teeth and denture use (ref: > 20 teeth)		
1–20 teeth, with denture	2.35 (2.33–2.36)	< .001
1–20 teeth, without denture	2.50 (2.48–2.51)	< .001
Edentulous, with denture	2.27 (2.26–2.29)	< .001
Edentulous, without denture	3.81 (3.79-3.84)	< .001
Oral health behavior		
Dental clinic visit (within 2 years) (ref: ≥ 1times)	1.65 (1.65–1.66)	< .001
Subjective oral health factors		
Chewing difficulty (ref: No)	3.22 (3.21–3.24)	< .001
Swallowing difficulty (ref: No)	1.58 (1.57–1.59)	< .001
Oral pain (ref: No)	2.57 (2.56–2.59)	< .001
Tooth sensitivity (ref: No)	2.39 (2.38–2.40)	< .001

*Note*. ref: reference; OR: odds ratio; CI: confidence interval; IADL: instrumental activities of daily living; BMI: body mass index; ^*^p < 0.05, ^**^p < 0.01, ^***^ *p* < 0.001

In the multivariate analysis adjusting for all variables (Model III), structural oral health factors showed the associations with frailty. In particular, being edentulous without denture use was associated with the highest odds of frailty (OR = 3.29, 95% CI = 3.27–3.32) and having fewer than 21 natural teeth without denture use was also significantly associated with frailty (OR = 2.07, 95% CI = 2.05–2.08). Among subjective oral health factors, swallowing difficulty (OR = 1.66, 95% CI = 1.65–1.66) and oral pain (OR = 1.59, 95% CI = 1.57–1.60) were associated with higher odds of frailty (Table 3).

**Table 3:** Perceived oral health as predictors of frailty among older adults (N = 3,729).

Variables	ModelⅠ	ModeⅡ	Model Ⅲ
	OR (95% CI)	OR (95% CI)	OR (95% CI)
General characteristics			
Age (years)	1.09 (1.09–1.09) ^***^	1.06 (1.06–1.06) ^***^	1.06 (1.06–1.06) ^***^
Education			
≤ 6 years (elementary school or less) (ref: > 6 years)	1.49 (1.48–1.50) ^***^	1.38 (1.38–1.39) ^***^	1.41 (1.41–1.42) ^***^
Living alone (ref: No)	1.05 (1.04–1.05) ^***^	1.16 (1.15–1.16) ^***^	1.11 (1.10–1.12) ^***^
Economic satisfaction	0.97 (0.97–.098) ^***^	0.98 (0.98–0.98) ^***^	0.98 (0.98–0.98) ^***^
Health-related factors			
Perceived health (ref: Good)		1.88 (1.86–1.89) ^***^	1.83 (1.82–1.85) ^***^
IADL (score)		1.16 (1.16–1.16) ^***^	1.16 (1.16–1.16) ^***^
Number of chronic diseases		1.23 (1.23–1.23) ^***^	1.25 (1.24–1.25) ^***^
BMI (kg/m^2^)		0.95 (0.95–0.95) ^**^	0.95 (0.95–0.95) ^***^
Oral health-related factors			
Structural factors			
Number of natural teeth and denture use (ref: > 20 teeth)			
1-20 teeth, with denture			0.96 (0.96–0.97) ^***^
1-20 teeth, without denture			2.07 (2.05–2.08) ^***^
Edentulous, with denture			0.75 (0.74–0.76) ^***^
Edentulous, without denture			3.29 (3.27–3.32) ^***^
Oral health behavior			
Dental clinic visit (within 2years) (ref: ≥ 1times)			1.34 (1.34–1.35) ^***^
Subjective oral health factors			
Chewing difficulty (ref: No)			1.29 (1.28–1.30) ^***^
Swallowing difficulty (ref: No)			1.66 (1.65–1.66) ^***^
Oral pain (ref: No)			1.59 (1.57–1.60) ^***^
Tooth sensitivity (ref: No)			1.29 (1.28–1.30) ^***^

*Note*. Model Ⅰ: included general characteristics; Model Ⅱ: additionally included health-related factors in Model Ⅰ; Model Ⅲ: additionally included oral health-related factors in Model Ⅱ; ref: reference; OR: odds ratio; CI: confidence interval; IADL: instrumental activities of daily living; BMI: body mass index; ^*^p < 0.05, ^**^p < 0.01, ^***^ *p* < 0.00

In the sex-stratified analysis, structural oral health factors showed the strongest association with frailty in both men and women. In particular, being edentulous without wearing dentures showed the highest odds of frailty in both women (OR = 3.76, 95% CI = 3.72–3.80) and men (OR = 2.95, 95% CI = 2.91–2.98). However, additional associated factors differed by sex. In women, poor perceived health status (OR = 3.35, 95% CI = 3.31–3.39) was associated with higher odds of frailty. In contrast, in men, oral pain (OR = 2.65, 95% CI = 2.62–2.69) was associated with higher odds of frailty. In addition, some variables showed opposite directions of association according to sex. For example, among participants with 1–20 natural teeth who wore dentures, the odds of frailty tended to decrease in women (OR = 0.89, 95% CI = 0.89–0.90), whereas they increased in men (OR = 1.05, 95% CI = 1.04–1.07). Similar sex differences were also observed in self-rated oral health factors: tooth sensitivity was associated with increased odds of frailty in women (OR = 1.82, 95% CI = 1.80–1.84), but with decreased odds of frailty in men (OR = 0.85, 95% CI = 0.84–0.86) (Table 4).

**Table 4:** Sex-stratified multivariate logistic regression analysis of factors associated with frailty (N = 3,729).

Variables	Women (n = 2,115)	Men (n = 1,614)
	OR (95% CI)	OR (95% CI)
General characteristics		
Age (years)	1.05 (1.05–1.06) ^***^	1.08 (1.08–1.09) ^***^
Education		
≤ 6 years (elementary school or less) (ref: > 6 years)	1.65 (1.64–1.66) ^***^	1.37 (1.36–1.38) ^***^
Living alone (ref: No)	1.24 (1.23–1.25) ^***^	1.14 (1.13–1.16) ^***^
Economic satisfaction	0.98 (0.98–0.99) ^***^	0.97 (0.96–0.97) ^***^
Health-related factors		
Perceived health (ref: Good)	3.35 (3.31–3.39) ^***^	1.14 (1.13–1.15) ^***^
IADL (score)	1.17 (1.17–1.17) ^***^	1.16 (1.15–1.16) ^***^
Number of chronic diseases	1.24 (1.23–1.24) ^***^	1.24 (1.24–1.25) ^***^
BMI (kg/m^2^)	0.95 (0.94–0.95) ^***^	0.95 (0.94–0.95) ^***^
Oral health-related factors		
Structural factors		
Number of natural teeth and denture use (ref: > 20 teeth)		
1–20 teeth, with denture	0.89 (0.89–0.90) ^***^	1.05 (1.04–1.07) ^***^
1–20 teeth, without denture	2.25 (2.23–2.27) ^***^	1.91 (1.88–1.93) ^***^
Edentulous, with denture	0.89 (0.88–0.90) ^***^	0.56 (0.55–0.57) ^***^
Edentulous, without denture	3.76 (3.72–3.80) ^***^	2.95 (2.91–2.98) ^***^
Oral health behavior		
Dental clinic visit (within 2years)	1.15 (1.14–1.16) ^***^	1.67 (1.66–1.69) ^***^
Subjective oral health factors		
Chewing difficulty (ref: No)	1.35 (1.33–1.36) ^***^	1.20 (1.18–1.21) ^***^
Swallowing difficulty (ref: No)	1.66 (1.65–1.67) ^***^	1.73 (1.72–1.75) ^***^
Oral pain (ref: No)	1.17 (1.16–1.18) ^***^	2.65 (2.62–2.69) ^***^
Tooth sensitivity (ref: No)	1.82 (1.80–1.84) ^***^	0.85 (0.84–0.86) ^***^

*Note*. ref: reference; OR: odds ratio; CI: confidence interval; IADL: instrumental activities of daily living; BMI: body mass index; ^*^p < 0.05, ^**^p < 0.01, ^***^ *p* < 0.001

In the sensitivity analysis, the results were generally consistent between the weighted and unweighted models. Although some factors were not statistically significant in the unweighted analysis, the overall findings remained consistent (S1–S4 Tables).

## Discussion

This study examined the associations of health-related and oral health-related factors with frailty among community-dwelling older adults and explored differences by sex. The results showed that various health factors were significantly associated with frailty, with the highest odds of frailty observed among edentulous individuals who did not use dentures. Furthermore, sex stratified analyses revealed that some oral health factors showed different or even opposite associations with frailty.

Our findings regarding general characteristics and health-related factors were consistent with those of previous studies [25,26]. Older age and living alone were associated with higher odds of frailty, whereas higher economic satisfaction was associated with lower odds of frailty. For those living alone, social vulnerability may increase due to greater loneliness and reduced social participation, which have been identified as important contributors to frailty [26]. Furthermore, a lower level of education was associated with higher odds of frailty; this finding is consistent with a previous study [27], which reported that individuals with higher education levels had a significantly lower risk of pre-frailty or frailty compared to those with lower education levels. Therefore, greater attention may be needed for vulnerable groups, such as individuals with lower education levels or those living alone.

This study found that higher economic satisfaction was associated with lower odds of frailty. This finding is consistent with a previous study [28], which reported that individuals living in socioeconomically deprived communities are more likely to experience moderate to severe frailty. Among health-related factors, functional dependency (IADL), the number of chronic diseases, and subjective health status were all significantly associated with frailty. In particular, subjective health status showed a strong association. This suggests that perceived health is an important indicator reflecting functional decline and overall health vulnerability [29]. Older adults who perceive their health negatively are more likely to experience reduced physiological reserve and increased functional dependence, and this association has been consistently reported in previous studies [30,31]. Therefore, subjective health may serve as a useful indicator for early identification of frailty in community-dwelling older adults.

Among structural oral health factors, the number of natural teeth and denture status were strongly associated with frailty, consistent with previous study [32]. In particular, the odds of frailty was highest among edentulous individuals who did not use dentures, which may be related to limitations in masticatory function that can influence dietary patterns and overall health status. A decrease in the number of natural teeth may be associated with restricted food choices and nutritional imbalances, which may reflect broader functional vulnerability rather than a single causal pathway [33]. On the other hand, denture use may provide some compensating for impaired masticatory function and may be associated with better function and nutritional status [4,34]. However, a systematic review examining the effects of prosthetic rehabilitation among older adults aged 60 years and older reported that, although prosthetic treatment significantly improved masticatory ability, nutritional status did not consistently improve in those who did not receive dietary counseling [35]. Therefore, this suggests the need for a multidisciplinary approach that goes beyond prosthetic treatment to mitigate the risks of poor nutritional status, frailty, and functional decline by improving oral health in older adults.

Subjective oral health factors also showed a significant association with frailty. Chewing and swallowing difficulties were associated with higher odds of frailty, suggesting that perceived oral functional limitations may serve as indicators of overall functional status rather than direct causal factors [36,37]. Furthermore, symptoms such as oral pain and tooth sensitivity may reflect an individual's perceived health status, highlighting that oral health captures multiple dimensions of health, including functional and psychosocial aspects, beyond simple structural conditions. This interpretation is consistent with the multidimensional nature of self-rated health [38].

In this study, the relationship between oral health and frailty differed by sex. Structural oral health factors were important in both men and women, but some differences were observed between the two groups. For instance, tooth sensitivity was associated with higher odds of frailty in women, whereas it was associated with lower odds in men. Likewise, having 1–20 natural teeth with denture use was associated with lower odds of frailty in women, whereas it was associated with higher odds in men. These findings suggest that the associations between oral health and frailty may differ by sex and are consistent with previous study reporting sex differences in the association between oral functional decline and frailty [8].

In particular, among women, sensory symptoms such as tooth sensitivity and subjective health perception were more closely associated with frailty. This may be because symptoms such as oral pain or discomfort are reflected in individuals’ perceptions of their health status and may be also related to sex differences in how health individual respond to health concern [14]. These findings are consistent with previous studies suggesting that health judgment is a complex cognitive process, as individuals evaluate their health by integrating various symptoms and experiences [38] with sex differences reported in the associations of socioeconomic and health-related factors with relf-rated health [39]. On the other hand, in men, more explicit symptom-based factors such as oral pain tended to be considered alongside structural oral conditions. This may be related to difference in the relatively importance placed on various health indicators when evaluating health [38]. Furthermore, even for the same oral symptoms, the way they are perceived and interpreted may differ by sex., and these differences may be related to sex disparities in health perception and healthcare utilization behaviors. Women may be more likely than men to seek healthcare for somatic symptoms [40]. In summary, the findings of this study suggest that oral health is associated with frailty through multiple interrelated dimensions, including structural conditions, functional limitations, and subjective perceptions, rather than through a single pathway [9]. In particular, the relative associations of these dimensions with frailty may vary by sex [7,8]. Therefore, a sex-sensitive approach should be considered in future strategies for promoting oral health and addressing frailty.

In this study, dental care utilization was found to be significantly associated with frailty, with a stronger association observed particularly in men. These results may reflect the greater burden of chronic conditions among individual with frailty [28] and sex differences in healthcare-seeking behavior [40] rather than dental care utilization itself being a direct cause of frailty. However, dental care utilization represents a potentially modifiable health behavior that may provide opportunities for oral health assessment and management [17]. If oral health problems are detected and managed early, this may provide an opportunity to maintain functional status and support overall health in later life [9], and issues such as mastication and swallowing are areas where early intervention is possible [41,42].

Therefore, an integrated approach may be beneficial, including regular assessment of oral health and efforts to promote dental care utilization among older adults through education and appropriate referrals. This aligns with recent international recommendations that integrate oral health into general health care and emphasize a prevention-centered approach [43]. Additionally, considering sex differences, it may be useful to consider function- and health awareness–focused approaches for women, and symptom-based approaches emphasizing early detection and referral for men [14,44].

This study has several limitations that should be considered. First, because we used secondary data, there were some constraints in how variables were defined and measured, and certain relevant factors may not have been included. Second, as most variables were based on self-reported data, the possibility of information bias remains. In particular, this study is limited by the fact that the oral health data were self-reported by participants during interviews conducted by trained survey interviewers and were not obtained through objective clinical assessments conducted by trained and standardized oral health examiners. In addition, although the survey included selected items from the K-GOHAI, the full instrument and its original scoring system were not used. Therefore, the findings cannot be interpreted as equivalent to standardized K-GOHAI scores. The oral health data in this study should thus be interpreted as limited indicators of participants’ perceived oral health status and functional discomfort rather than as measures of objective oral disease status or clinical diagnoses. Future studies should incorporate standardized oral examinations conducted by trained assessors together with validated oral health assessment instruments to improve measurement reliability and validity. Third, given the cross-sectional design of the study, it is difficult to draw clear conclusions about causal relationships. Fourth, this study used the frailty instrument developed by Kim and Sunwoo [22] for older Korean adults. However, the frailty phenotype proposed by Fried et al., which is widely used internationally, was not applied. Because the two instruments differ in their components and classification criteria, caution is required when directly comparing the prevalence and association of frailty observed in this study with findings from studies using the Fried phenotype. Accordingly, the frailty measure used in this study may not fully capture the construct represented by the Fried frailty phenotype. Future studies should apply both the Fried phenotype and a frailty instrument developed for the Korean population to examine their agreement and comparative predictive validity. Finally, as the study was conducted among community-dwelling older adults in Korea, the findings should be interpreted with caution when generalizing them to populations in other countries or cultural setting.

## Conclusion

This study found that structural oral health, oral health behaviors, and subjective oral health factors were all significantly associated with frailty in community-dwelling older adults. In particular, structural oral health factors were most strongly associated with frailty in both men and women, whereas some subjective oral health factors showed different or even opposite patterns of association by sex. Furthermore, health behaviors such as dental care utilization were also associated with frailty. Therefore, oral health should be considered in relation to frailty among older adults, taking into account sex differences and behavioral factors.

## Supporting information

S1 Table General characteristics of the study participants. (DOCX)

S2 Table Univariate logistic regression analysis for factors associated with frailty. (DOCX)

S3 Table Perceived oral health as predictors of frailty among older adults. (DOCX)

S4 Table Sex-stratified multivariate logistic regression analysis of factors associated with frailty. (DOCX)
