# Supplementary material for: Multidimensional oral health and frailty in older adults: A sex-stratified cross-sectional study
Source: PLoS One. 2026 Oct 16;21(10):e0360682. doi: 10.1371/journal.pone.0360682 (PMC13674771; doi:10.1371/journal.pone.0360682)
Supplement: Supplementary file 1 [file pone.0360682.s001.docx]

S1 Table. General characteristics of the study participants	(N = 3,729	)

| Variables | n (%) or M ± SD | Actual range |
| --- | --- | --- |
| General characteristics |  |  |
| Age (years) | 75.23 ± 7.11 | 65–100 |
| Sex |  |  |
| Female | 2,115 (56.7) |  |
| Male | 1,614 (43.3) |  |
| Education |  |  |
| 0–6 | 1,590 (42.6) |  |
| 7~ | 2,139 (57.4) |  |
| Living alone |  |  |
| Yes | 801 (21.5) |  |
| No | 2,928 (78.5) |  |
| Economic satisfaction | 56.72 ± 18.02 |  |
| Health-related factor |  |  |
| Perceived health |  |  |
| Good | 831 (22.3) |  |
| Poor | 2,898 (77.7) |  |
| IADL (score) | 0.48 ± 1.56 | 0–10 |
| Number of chronic diseases | 1.72 ± 1.30 | 0–7 |
| BMI (kg/m^2^) | 23.61 ± 2.65 |  |
| Oral health-related factors |  |  |
| Structural factors |  |  |
| Number of natural teeth and denture use |  |  |
| >20 teeth | 2,329 (62.5) |  |
| 1-20 teeth, with denture | 400 (10.7) |  |
| 1-20 teeth, without denture | 430 (11.5) |  |
| Edentulous, with denture | 316 (8.5) |  |
| Edentulous, without denture | 228 (6.1) |  |
| Oral health behavior |  |  |
| Dental clinic visit (within 2years) |  |  |
| Yes | 1,081 (29.0) |  |
| No | 2,646 (71.0) |  |
| Subjective oral health factors |  |  |
| Chewing difficulty |  |  |
| Yes | 1,211 (32.5) |  |
| No | 2,518 (67.5) |  |
| Swallowing difficulty |  |  |
| Yes | 1,163 (31.2) |  |
| No | 2,566 (68.8) |  |
| Oral pain |  |  |
| Yes | 333 (8.9) |  |
| No | 3,396 (91.1) |  |
| Tooth sensitivity |  |  |
| Yes | 529 (14.2) |  |
| No | 3,200 (85.8) |  |
| Frailty status |  |  |
| Robust | 1,960 (52.5) |  |
| Pre-frail | 1,174 (31.5) |  |
| Frail | 595 (16.0) |  |

SD: standard deviation; IADL: Instrumental activities of daily living; BMI: body mass index; missing data n (%) : BMI:24(0.6%); Visit dental clinic (within 2 years): 2(0.1%); Number of natural teeth 26(0.7%)
