# Supplementary material for: Multidimensional oral health and frailty in older adults: A sex-stratified cross-sectional study
Source: PLoS One. 2026 Oct 16;21(10):e0360682. doi: 10.1371/journal.pone.0360682 (PMC13674771; doi:10.1371/journal.pone.0360682)
Supplement: Supplementary file 2 [file pone.0360682.s002.docx]

S2 Table. Univariate logistic regression analysis for factors associated with frailty	(N = 3,729)

| Variables | OR (95% CI) | *p*-value |
| --- | --- | --- |
| General characteristics |  |  |
| Age (years) | 1.12 (1.10–1.13) | < .001 |
| Sex |  |  |
| Female (ref: Male) | 1.03 (0.98–1.07) | .258 |
| Education |  |  |
| ≤ 6 years (elementary school or less) (ref: > 6 years) | 2.60 (2.17–3.12) | < .001 |
| Economic satisfaction | 0.98 (0.96–0.97) | < .001 |
| Living alone (ref: No) | 1.81 (1.49–3.20) | < .001 |
| Health-related factors |  |  |
| Perceived health (ref: Good) | 3.71 (2.74–5.02) | < .001 |
| IADL (score) | 1.37 (1.30–1.43) | < .001 |
| Number of chronic diseases | 1.40 (1.32–1.50) | < .001 |
| BMI (kg/m^2^) | 0.92 (0.89–0.96) | < .001 |
| Oral health-related factors |  |  |
| Structural factors |  |  |
| Number of natural teeth and denture use (ref: > 20 teeth) |  |  |
| 1–20 teeth, with denture | 2.25 (1.72–2.95) | < .001 |
| 1–20 teeth, without denture | 2.30 (1.77–2.99) | < .001 |
| Edentulous, with denture | 2.26 (1.68–3.04) | < .001 |
| Edentulous, without denture | 4.13 (3.05–5.59) | < .001 |
| Oral health behavior |  |  |
| Dental clinic visit (within 2 years) (ref: ≥ 1times) | 1.67 (1.35–2.06) | < .001 |
| Subjective oral health factors |  |  |
| Chewing difficulty (ref: No) | 3.21 (2.62–3.92) | < .001 |
| Swallowing difficulty (ref: No) | 1.49 (1.24–1.78) | < .001 |
| Oral pain (ref: No) | 2.35 (1.82–3.04) | < .001 |
| Tooth sensitivity (ref: No) | 2.20 (1.77–2.73) | < .001 |

ref: reference; OR: odds ratio; CI: confidence interval; IADL: Instrumental activities of daily living; BMI: body mass index
