# Supplementary material for: Multidimensional oral health and frailty in older adults: A sex-stratified cross-sectional study
Source: PLoS One. 2026 Oct 16;21(10):e0360682. doi: 10.1371/journal.pone.0360682 (PMC13674771; doi:10.1371/journal.pone.0360682)
Supplement: Supplementary file 3 [file pone.0360682.s003.docx]

S3 Table. Perceived oral health as predictors of frailty among older adults	(N=3,729)

| Variables | Model Ⅰ | Model Ⅱ | Model Ⅲ |
| --- | --- | --- | --- |
|  | OR (95% CI) | OR (95% CI) | OR (95% CI) |
| General characteristics |  |  |  |
| Age (years) | 1.10 (1.08–1.12) ^***^ | 1.07 (1.50–1.08) ^***^ | 1.07 (1.05–1.08) ^***^ |
| Education |  |  |  |
| ≤ 6 years (elementary school or less) (ref: > 6 years) | 1.37 (1.13–1.71) ^***^ | 1.32 (1.07–1.63) ^***^ | 1.33 (1.08–1.65) ^**^ |
| Living alone (ref: No) | 0.99 (0.79–1.24) | 1.09 (0.87–1.37) | 1.08 (0.85–1.37) |
| Economic satisfaction | 0.97 (0.97–0.98) ^***^ | 0.98 (0.97–0.98) ^***^ | 0.98 (0.97–0.98) ^***^ |
| Health related factors |  |  |  |
| Perceived health (ref: Good) |  | 1.92 (1.39–2.65) ^***^ | 1.87 (1.34–2.60) ^***^ |
| IADL (score) |  | 1.17 (1.11–1.23) ^***^ | 1.17 (1.11–1.24) ^***^ |
| Number of chronic diseases |  | 1.18 (1.10–1.28) ^***^ | 1.20 (1.16–1.35) ^***^ |
| BMI (kg/m^2^) |  | 0.95 (0.92–0.98) ^**^ | 0.95 (0.91–0.98) ^**^ |
| Oral health related factors |  |  |  |
| Structural factors |  |  |  |
| Number of natural teeth and denture use (ref: > 20 teeth) |  |  |  |
| 1-20 teeth, with denture |  |  | 0.98 (0.72–1.35) |
| 1-20 teeth, without denture |  |  | 1.83 (1.36–2.45) ^***^ |
| Edentulous, with denture |  |  | 0.83 (0.59–1.18) |
| Edentulous, without denture |  |  | 3.50 (2.47–4.96) ^***^ |
| Oral health behavior |  |  |  |
| Dental clinic visit  (within 2 years) |  |  | 1.58 (1.11–1.29) ^***^ |
| Subjective oral health factors |  |  |  |
| Chewing difficulty (ref: No) |  |  | 1.41 (1.08–1.86) ^*^ |
| Swallowing difficulty (ref: No) |  |  | 1.55 (1.25–1.91) ^***^ |
| Oral pain (ref: No) |  |  | 1.51 (1.07–2.13) ^*^ |
| Tooth sensitivity (ref: No) |  |  | 1.12 (0.82–1.52) |

Model Ⅰ: included general characteristics; Model Ⅱ: additionally, included health related factors in Model Ⅰ; Model Ⅲ: additionally, included oral health-related factors in Model Ⅱ; ref: reference; OR: odds ratio; CI: confidence interval; IADL: Instrumental activities of daily living; BMI: body mass index; ^*^*p* < 0.05, ^**^*p* < 0.01, ^***^ *p* < 0.001
