# Supplementary material for: Multidimensional oral health and frailty in older adults: A sex-stratified cross-sectional study
Source: PLoS One. 2026 Oct 16;21(10):e0360682. doi: 10.1371/journal.pone.0360682 (PMC13674771; doi:10.1371/journal.pone.0360682)
Supplement: Supplementary file 4 [file pone.0360682.s004.docx]

S4 Table. Sex-stratified multivariate logistic regression analysis of factors associated with frailty	(N=3,729	)

| Variables | Women (n = 2,115) | Men (n = 1,614) |
| --- | --- | --- |
|  | OR (95% CI) | OR (95% CI) |
| General characteristics |  |  |
| Age (year) | 1.05 (1.03-1.07) ^***^ | 1.09 (1.06-1.12) ^***^ |
| Education |  |  |
| ≤ 6 years (elementary school or less) (ref: > 6 years) | 1.52 (1.11-2.07) ^**^ | 1.36 (0.97-1.92) |
| Living alone (ref: No) | 1.18 (0.89-1.57) | 1.21 (0.72-2.01) |
| Economic satisfaction | 0.98 (0.98-1.57) ^***^ | 0.97 (0.96-0.98) ^***^ |
| Health-related factors |  |  |
| Perceived health (ref: Good) | 3.15 (1.86-5.32) ^***^ | 1.19 (0.76-1.86) |
| IADL (score) | 1.17 (1.09-1.26) ^***^ | 1.18 (1.09-1.28) ^***^ |
| Number of chronic diseases | 1.22 (1.10-1.35) ^***^ | 1.15 (1.02-1.30) ^*^ |
| BMI (kg/m^2^) | 0.94 (0.90-0.98) ^**^ | 0.95 (1.02-1.30) |
| Oral health-related factors |  |  |
| Structural factors |  |  |
| Number of natural teeth and denture use (ref: >20 teeth) |  |  |
| 1-20 teeth, with denture | 0.08 (0.52-1.24) | 1.23 (0.76-1.99) |
| 1-20 teeth, without denture | 2.09 (1.44-3.03) ^***^ | 1.47 (0.90-2.40) |
| Edentulous, with denture | 0.97 (0.62-1.52) | 0.62 (0.36-1.09) |
| Edentulous, without denture | 3.95 (2.48-6.30) ^***^ | 3.05 (1.79-5.22) ^***^ |
| Oral health behavior |  |  |
| Visit dental clinic (within 2year) | 1.15 (0.84-1.58) | 1.72 (1.17-2.54) ^**^ |
| Subjective oral health factors |  |  |
| Chewing difficulty (ref: No) | 1.49 (1.04-2.12) ^*^ | 1.33 (0.85-1.06) |
| Swallowing difficulty (ref: No) | 1.58 (1.20-2.08) ^**^ | 1.56 (1.12-2.18) ^**^ |
| Oral pain (ref: No) | 1.21 (0.78-1.87) | 2.24 (1.26-4.01) ^**^ |
| Tooth sensitivity (ref: No) | 1.43 (0.96-2.13) | 0.82 (0.50-1.36) |

ref: reference; OR: odds ratio; CI: confidence interval; IADL: instrumental activities of daily living; BMI: body mass index; ^*^*p* < 0.05, ^**^*p* < 0.01, ^***^ *p* < 0.001
